# Coverage, completion and outcomes of COVID-19 risk assessments in a multi-ethnic nationwide cohort of UK healthcare workers: a cross-sectional analysis from the UK-REACH Study

**DOI:** 10.1136/oemed-2022-108700

**Published:** 2023-05-23

**Authors:** Christopher A Martin, Katherine Woolf, Luke Bryant, Charles Goss, Mayuri Gogoi, Susie Lagrata, Padmasayee Papineni, Irtiza Qureshi, Fatimah Wobi, Laura Nellums, Kamlesh Khunti, Manish Pareek

**Affiliations:** 1 Department of Respiratory Sciences, University of Leicester, Leicester, UK; 2 Department of Infection and HIV Medicine, University Hospitals of Leicester NHS Trust, Leicester, UK; 3 Research Department of Medical Education, University College London Medical School, London, UK; 4 Department of Occupational Health, University Hospitals of Leicester NHS Trust, Leicester, UK; 5 Queen Square Insitute of Neurology and National Hospital for Neurology and Neurosurgery, University College London Hospitals NHS Foundation Trust, London, UK; 6 Department of Infectious Diseases, London North West University Healthcare NHS Trust, Harrow, UK; 7 Population and Lifespan Sciences, University of Nottingham, Nottingham, UK; 8 Public Health Institute, Liverpool John Moores University, Liverpool, UK; 9 School of Law, University of Leicester, Leicester, UK; 10 Diabetes Research Centre, University of Leicester, Leicester, UK

**Keywords:** COVID-19, Risk assessment, Ethnic Groups, Health Personnel

## Abstract

**Introduction:**

There are limited data on the outcomes of COVID-19 risk assessment in healthcare workers (HCWs) or the association of ethnicity, other sociodemographic and occupational factors with risk assessment outcomes.

**Methods:**

We used questionnaire data from UK-REACH (UK Research study into Ethnicity And COVID-19 outcomes in Healthcare workers), an ethnically diverse, nationwide cohort of UK HCWs. We derived four binary outcomes: (1) offered a risk assessment; (2) completed a risk assessment; (3) working practices changed as a result of the risk assessment; (4) wanted changes to working practices after risk assessment but working practices did not change.

We examined the association of ethnicity, other sociodemographic/occupational factors and actual/perceived COVID-19 risk variables on our outcomes using multivariable logistic regression.

**Results:**

8649 HCWs were included in total. HCWs from ethnic minority groups were more likely to report being offered a risk assessment than white HCWs, and those from Asian and black ethnic groups were more likely to report having completed an assessment if offered. Ethnic minority HCWs had lower odds of reporting having their work change as a result of risk assessment. Those from Asian and black ethnic groups were more likely to report no changes to their working practices despite wanting them.

Previous SARS-CoV-2 infection was associated with lower odds of being offered a risk assessment and having adjustments made to working practices.

**Discussion:**

We found differences in risk assessment outcomes by ethnicity, other sociodemographic/occupational factors and actual/perceived COVID-19 risk factors. These findings are concerning and warrant further research using actual (rather than reported) risk assessment outcomes in an unselected cohort.

**WHAT IS ALREADY KNOWN ON THIS TOPIC**

- Ethnic minority groups and healthcare workers have been disproportionately impacted by the COVID-19 pandemic. National Health Service (NHS) leaders were advised to take account of ethnic minority in COVID-19 risk assessments in April 2020 and were instructed to complete risk assessment of all ethnic minority staff by July 2020. However, only 61% of acute care trusts had completed the risk assessment of ethnic minority staff by this time. A survey study of UK doctors in February 2021 found that only 55% reported that they had been risk assessed and were confident that necessary adjustments were made.

**WHAT THIS STUDY ADDS**

- In this nationwide study in a highly ethnically diverse cohort of NHS workers, we found that the majority of staff reported having been risk assessed. Ethnic minority healthcare workers were more likely than those from white ethnic groups to report having been offered a COVID-19 risk assessment and more likely to have completed a risk assessment once offered. However, ethnic minority healthcare workers were less likely to report having changes made to working practices after risk assessment and more likely to have unfulfilled wishes for changes to working practices.

**HOW THIS STUDY MIGHT AFFECT RESEARCH, PRACTICE OR POLICY**

- This is the largest study of risk assessment outcomes in UK healthcare workers. We highlight differences in self-reported risk assessment outcomes by ethnicity and by other sociodemographic, occupational and clinical parameters. These findings are concerning and warrant urgent further investigation in an unselected cohort to inform future policy around COVID-19 risk assessment, thus protecting the healthcare workforce and preventing the ethnic disparities of the COVID-19 pandemic from widening.

## Introduction

It has been established that working as a healthcare worker (HCW) represents a risk factor for infection with SARS-CoV-2 when compared with the general population.^1^ Ethnic minority groups in the UK and the USA are also at higher risk of infection with SARS-CoV-2 than white groups and may also be at higher risk of adverse outcome from COVID-19.^2–4^ In studies examining infection risk in HCW cohorts, those from ethnic minority groups have been demonstrated to be at higher risk of infection, implying that risk is compounded in ethnic minority HCWs.^1 5–7^ There is a wealth of evidence to suggest that the increased risk of COVID-19 faced by ethnic minority groups is underpinned by social, economic and health inequalities.^2 8–10^

In recognition of this increased risk, in March 2020, National Health Service (NHS) leaders were instructed to make adjustments for vulnerable staff.^11^ Further communications in April identified ethnic minority as an emerging risk factor for COVID-19 and recommended that this was taken account of when deciding whether adjustments should be made to working practices in order to safeguard against this risk.^12^ This was followed in June by communications mandating that risk assessments for staff in at-risk groups be completed within a month.^13^ Despite such communications, it was reported that only 61% of acute care trusts had completed the assessment of ethnic minority staff by July 2020.^13^ A survey of British Medical Association members in February 2021 found that only 55% of around 7000 respondents reported that they had been risk assessed and were confident that necessary adjustments were made.^14^

Guidance is available on how to conduct COVID-19 risk assessments including assessment of workplace, workforce and individual vulnerability factors.^15^ Recommendations are that assessment of individual vulnerability should be conducted by conversation with a manager/supervisor or health and safety representative and should take account of age, sex, long-term health conditions, ethnicity, pregnancy and, more recently, SARS-CoV-2 vaccination status. Furthermore, it is advised that assessments take account of psychological and social factors, risk behaviours and mental well-being.^15 16^

Despite the importance of individual COVID-19 risk assessment for HCWs, there is little evidence on the proportion of HCWs who have undergone risk assessment or whether changes were made to working practices as a result. Furthermore, since the introduction of this national policy, there has been no systematic evidence on whether the likelihood of being risk assessed or having amendments made to working practices differs according to ethnicity and other sociodemographic and occupational factors. To address this knowledge gap, we analysed data from the UK-REACH (UK Research study into Ethnicity And COVID-19 outcomes in Healthcare workers) Study.

## Methods

This work uses data from the baseline questionnaire of the UK-REACH cohort study (administered December 2020–March 2021). The cohort includes HCWs aged 16 years or older. Recruitment and study methods are described in detail in online supplemental text 1, online supplemental figure 1 and previous work.^5 17–19^

10.1136/oemed-2022-108700.supp1 Supplementary data

**Figure 1:**
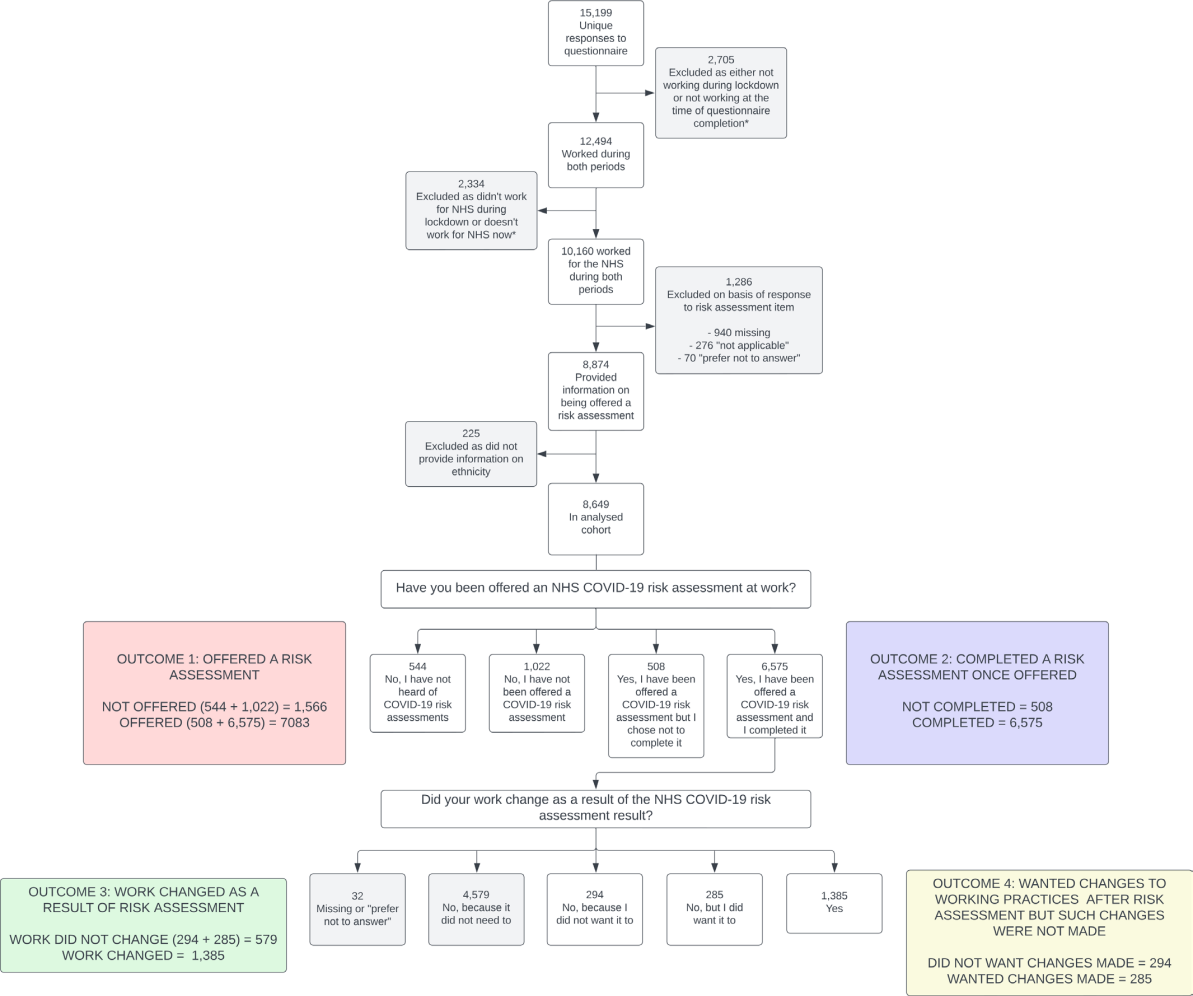
Formation of the analysed sample and derivation of outcome measures. *at time of questionnaire completion. NHS, National Health Service.

Formation of the analysed sample is shown in figure 1. The questionnaire items specifically asked about NHS COVID-19 risk assessments; therefore, participants who did not report working for the NHS were excluded from the analyses. Participants who indicated they were not working at the time of questionnaire completion were not asked the risk assessment questions and were excluded from the analyses. To ensure our measures of occupational exposure to COVID-19 reflected levels of exposure at the time of risk assessment roll-out, we used answers to questions about occupational circumstances in the weeks following the first UK national lockdown (this period was referred to throughout the questionnaire as ‘the UK national lockdown on 23 March 2020’). Those with missing ethnicity data and those not working during lockdown were excluded. Further exclusions were dependent on the outcome of the analysis (figure 1).

We derived four binary outcome measures from two questionnaire items (figure 1). Outcomes 1 and 2 are derived from the question ‘Have you been offered an NHS COVID-19 risk assessment at work?’. Outcomes 3 and 4 are derived from the question ‘Did your work change as a result of the NHS COVID-19 risk assessment result?’, which was asked only of those who indicated they had completed a COVID-19 risk assessment.

Outcome 1: offered a risk assessment (not offered vs offered); outcome 2: completed a risk assessment when offered (completed vs not completed) but excludes those not offered; outcome 3: working practices changed as a result of the risk assessment (changed vs did not change); outcome 4: wanted changes to working practices after risk assessment but working practices did not change (did not want change vs wanted change).

For analyses using outcomes 3 and 4, we only included those who indicated the assessment result could, or should, have led to workplace adjustments (ie, we excluded those who responded ‘No [my work did not change] because it did not need to’).

Our primary exposure of interest was self-reported ethnicity. To maximise statistical power, we categorised ethnicity into the five broad ethnic groups (white, Asian, black, mixed and other) suggested by the UK Office for National Statistics.^20^

Potential confounders of the relationship between ethnicity and our outcome measures were hypothesised to be: demographic characteristics (age, sex); occupational group (categorised into medical, nursing, allied health professional (AHP), dental and administrative/estates/other); migration status (categorised as born in the UK and born overseas).

We also explored the effects of other variables related to the risk, or perceived risk, of severe COVID-19 and perceived risk of transmitting COVID-19 to others on the outcome measures. These variables are as follows (for details, see online supplemental table 1): level of exposure to patients with COVID-19 during lockdown; number of long-term physical health conditions; body mass index; previous SARS-CoV-2 infection status; perceived risk of hospitalisation with COVID-19; perceived risk of unknowingly spreading COVID-19; cohabiting with someone over the age of 65 years.

It should be noted that occupation might be considered to be a mediator rather than a confounder of the relationship between ethnicity and risk assessment outcome. However, occupation may have influenced inclusion of HCWs from ethnic minority groups in the cohort;^21^ therefore, we elected to adjust for occupation when examining ethnic differences in multivariable models.

In an analysis of two subgroups, we investigated the effect of specific occupational parameters on outcomes 1 and 2, namely pay band for those on the NHS agenda for change pay scales and grade for doctors. The agenda for change pay scales determines the salary of NHS staff other than doctors, dentists and those in very senior management positions. There are nine bands (band 1–band 9) and salary increases as band increases. In this analysis, we use this scale as a proxy measure for occupational seniority. We did not conduct the same analysis on outcomes 3 and 4 as it was felt that the number of participants included in such analyses would be too small to provide meaningful results. We excluded those who did not provide information on grade or NHS pay band from the relevant subgroup analysis.

We summarised categorical variables as frequency and percentage, and continuous variables as median and IQR.

We used multivariable logistic regression to determine associations of the variables described above with the outcomes and present adjusted ORs (aORs), 95% CIs and p values. Ethnicity and hypothesised confounders (age, sex, occupation and migration status) were included in a base model. We then added the variables relating to risk/perceived risk of COVID-19 to this base model separately such that the aORs included in the figure are adjusted for the variables in the base model (but not the other risk/perceived risk variables). We did not include the ‘perceived risk’ variables in the analysis of outcome 1 as we felt these would not have influenced whether an HCW was offered a risk assessment.

Multiple imputation was used to impute missing data in logistic regression models. The imputation models contained all variables used in the analysis except the one being imputed. Rubin’s Rules were used to combine the parameter estimates and SEs from 10 imputations into a single set of results.^22^

We conducted multiple sensitivity analyses: (1) analysis of the main outcome measures in complete cases to test the effect multiple imputation had on results; (2) examination of univariable ORs for the association of ethnicity with our outcome measure to test the impact of adjustment for other variables in the base model; (3) an analysis which excludes those with no direct contact with patients with COVID-19 to test the hypothesis that differences in exposure to patients with COVID-19 by ethnicity may have influenced ethnic differences in risk assessment coverage and outcome.

We set statistical significance at p<0.05 and did not correct for multiple comparisons because of lower statistical power due to smaller sample sizes in analyses using outcomes 3 and 4, and because we felt it would be overly restrictive for this exploratory analysis.

All analyses were conducted using Stata V.17. Figures were created using GraphPad Prism.

For details on public/professional involvement and engagement, see online supplemental text 2.

The funders had no role in study design, data collection, data analysis, interpretation or writing of the report.

## Results

Participants included and excluded in each analysis are detailed in figure 1. A description of the analysed cohort and the amount of missing data for each variable is shown in table 1. Of the 8649 HCWs included, 1820 (21.0%) were from Asian, 371 (4.3%) from black, 360 (4.2%) from mixed and 198 (2.3%) from other ethnic groups. One thousand eight hundred eighty-two (21.8%) had missing data in at least one of the variables of interest.

**Table 1:** Description of the analysed cohort

Variable	Description N=8649
Ethnicity	
White Asian Black Mixed Other	5900 (68.2) 1820 (21.0) 371 (4.3) 360 (4.2) 198 (2.3)
Age in years, median (IQR)	44 (34–53)
Missing	43 (0.5)
Sex	
Male Female Missing	2202 (25.5) 6429 (74.3) 18 (0.2)
Occupation	
Medical/medical support Nursing (inc. midwives, nursing associates) Allied health professionals* Dental Administrative/estates/other Missing	2366 (27.4) 1903 (22.0) 3360 (38.9) 289 (3.3) 446 (5.2) 285 (3.3)
Migration status	
Born in the UK Born overseas Missing	6249 (72.3) 2379 (27.5) 21 (0.2)
Exposure to patients with COVID-19 during lockdown	
None (or remote contact only) Face-to-face with social distancing only Physical contact Missing	4122 (47.7) 520 (6.0) 3938 (45.5) 69 (0.8)
Number of long-term physical health conditions†	
0 1 ≥2 Missing	5819 (67.3) 1721 (19.9) 419 (4.8) 690 (8.0)
Body mass index (kg/m^2^)	
<25 ≥25 and <30 ≥30 Missing	3675 (42.5) 2360 (27.3) 1513 (17.5) 1101 (12.7)
Perceived risk of being hospitalised with COVID-19 in the next 6 months (scale 0–100), median (IQR)	20 (5–50)
Missing	534 (6.2)
Level of concern about unknowingly spreading COVID-19	
Not at all concerned A little concerned Quite concerned Very concerned Missing	1071 (12.4) 3023 (35.0) 2197 (25.4) 1891 (21.9) 467 (5.4)
SARS-CoV-2 infection status (on 1 May 2020)	
Uninfected Infected Missing	7075 (81.8) 1068 (12.4) 506 (5.9)
Cohabitation with those over 65 years old	
Does not live with someone over the age of 65 Lives with someone over the age of 65 Missing	7842 (90.7) 591 (6.8) 216 (2.5)

Table 1 provides a description of the 8649 HCWs who worked for the NHS during lockdown and at the time of questionnaire response, provided information on their ethnicity and answered the question about being offered risk assessments. All data in the right-hand column are n (%) unless stated otherwise.

*Include pharmacists, health scientists, ambulance workers and those in optical roles.

†Include diabetes, heart disease, hypertension, previous stroke, kidney or liver disease, asthma, lung condition other than asthma, cancer, neurological disease, organ transplant and immunosuppression.

HCWs, healthcare workers; NHS, National Health Service.

Overall, 81.9% (n=8649) reported being offered a risk assessment (outcome 1) and 92.8% (n=7083) reported completing a COVID-19 risk assessment once offered (outcome 2). Among those who completed a risk assessment and did not indicate that workplace adjustments were unnecessary, 70.5% (n=1964) reported having such amendments made (outcome 3). In those who reported their work did not change as a result of risk assessment and who did not indicate it did not need to change, half (49.2%, n=579) reported unfulfilled wishes for workplace adjustments (outcome 4).

A description of the cohort stratified by responses to the two questionnaire items can be found in online supplemental text 3 and online supplemental tables 2 and 3.

**Figure 2:**
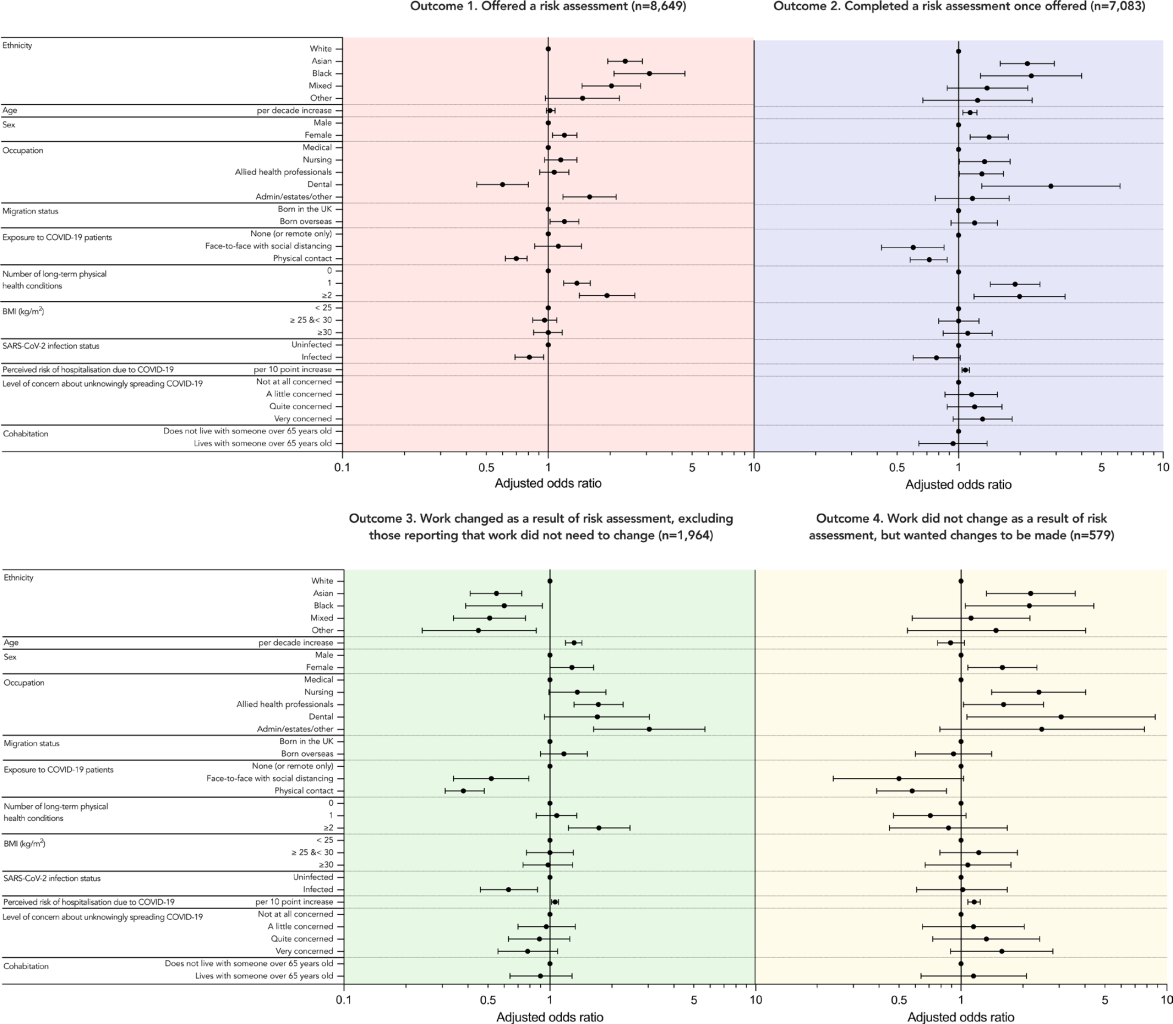
Adjusted ORs and 95% CIs are shown for the association of sociodemographic, occupational and perceived risk variables with four outcomes relating to NHS COVID-19 risk assessments derived from multivariable logistic regression. ORs are adjusted for age, sex, ethnicity, occupation and migration status. All outcome variables are binary and all analyses include only HCWs who were working for the NHS during UK national lockdown and at the time of questionnaire completion. Outcome 1 is whether or not an HCW was offered a COVID-19 risk assessment; the analysed sample includes all HCWs who provided information on their ethnicity and the outcome of interest. Certain risk perception variables were not included in this analysis (as shown by the lack of ORs) as it was felt that these were unlikely to influence being offered a risk assessment. Outcome 2 is whether or not an HCW chose to complete a risk assessment; the analysed sample includes all those in the previous analysis who were offered a risk assessment. Outcome 3 is whether or not changes were made to the working practices of HCWs after the risk assessment; the analysed sample includes HCWs who completed a risk assessment and provided information on the outcome with those indicating that work did not need to change excluded. Outcome 4 is whether or not an HCW wanted changes to be made at work; the analysed sample includes all HCWs who indicated their work did not change, excluding those who indicated that their work did not need to change. Derivation of all outcomes is described in detail in figure 1. In the occupation variable, nursing includes midwives, nursing associates and healthcare assistants; allied health professionals include pharmacists, healthcare scientists, ambulance workers and those in optical roles. BMI, body mass index; HCWs, healthcare workers; NHS, National Health Service.

Figure 2 and online supplemental tables 4 and 5 show the results of the logistic regression analyses for outcomes 1–4.

Compared with white HCWs, and after adjustment for age, sex occupation and migration status, HCWs from Asian, black and mixed ethnic groups were more likely to report having been offered a risk assessment, and those from Asian and black ethnic groups were more likely to report having completed an assessment if offered. Among those who completed an assessment, HCWs from all ethnic minority groups had lower odds of reporting having their work change as a result of risk assessment, and those from Asian and black ethnic groups were more likely to report no change to their working practices despite wanting it.

Those in dental roles had lower odds than those in medical roles of reporting being offered a risk assessment but higher odds of reporting completing the assessment once offered. Those in AHP roles and those in ‘administrative/estates/other’ roles were more likely than those in medical roles to report having adjustments made to working practices. Those in nursing, AHP and dental roles were more likely to report not having changes to their working practices despite wanting them.

An increasing number of long-term conditions was positively associated with being offered, completing and having work changed as a result of risk assessment.

HCWs who reported physical contact with patients with COVID-19 during lockdown were less likely than those who did not report being offered, having completed and not having work changed as a result of risk assessment. They were also less likely to want changes but not get them.

A history of SARS-CoV-2 infection was associated with lower odds of being offered a risk assessment and having changes made to working practices.

Among those on the NHS agenda for change pay scales, those in band 1 or 2 were less likely to report being offered a risk assessment than those in band 5. With each band above 5, the OR for reporting being offered a risk assessment increased (table 2).

**Table 2:** Association of agenda for change pay band with being offered and completing a risk assessment in a cohort of non-medical staff

	Outcome 1: offered a risk assessment (N=5798) aOR (95% CI)	Outcome 2: completed a risk assessment (N=4736) aOR (95% CI)
Band 1 or 2	0.58 (0.37 to 0.89)	0.60 (0.27 to 1.32)
Band 3 or 4	1.05 (0.79 to 1.41)	1.19 (0.67 to 2.10)
Band 5	Ref	Ref
Band 6	1.27 (1.06 to 1.52)	1.01 (0.73 to 1.40)
Band 7	2.11 (1.71 to 2.61)	1.04 (0.73 to 1.49)
Band 8 and above	3.00 (2.29 to 3.93)	0.76 (0.52 to 1.10)

aOR, adjusted OR.

**Table 3:** Association of grade with being offered and completing a risk assessment in a cohort of medical staff

	Outcome 1: offered a risk assessment (N=2332) aOR (95% CI)	Outcome 2: completed a risk assessment (N=1969) aOR (95% CI)
Foundation trainee	0.69 (0.38 to 1.25)	0.86 (0.38 to 1.94)
Core trainee	0.57 (0.33 to 0.97)	0.85 (0.39 to 1.83)
Specialty trainee	0.71 (0.48 to 1.05)	1.14 (0.65 to 2.00)
Consultant	Ref	Ref
General practitioner	0.36 (0.27 to 0.49)	2.06 (1.17 to 3.64)
Other	0.73 (0.27 to 1.95)	0.55 (0.18 to 1.67)

ORs in tables 2 and 3 are adjusted for age, sex, ethnicity and migration status. As agenda for change pay band increases so does salary. Band 5 is the level of a newly qualified nurse. In the analysis of medical staff, a foundation trainee is a newly qualified doctor in the first 2 years of training after medical school; a core trainee has completed foundation training and selected a broad area of specialisation (such as acute care, medicine, surgery or psychiatry) but not yet started specialty training; a specialty trainee (otherwise known as a registrar) has completed core training and is undertaking training in a particular specialty area; a consultant has completed training in a particular specialty; a general practitioner is a doctor who has completed training in general practice and typically works in the community rather than in hospital.

aOR, adjusted OR.

Among doctors, general practitioners had around one-third of the odds of reporting being offered a risk assessment than consultants but were more likely to report completing a risk assessment once offered (table 3).

In an analysis of complete cases, significant findings were largely unchanged (online supplemental tables 6 and 7). Significant findings change very little between the unadjusted and adjusted models with the exception of outcome 4 where ethnic differences were attenuated in the unadjusted model (online supplemental table 8). This may be due to differences in the occupational and sex distributions across ethnic groups (shown previously^5^). In an analysis that excludes those without direct contact with a patient with COVID-19, significant findings relating to ethnicity are largely unchanged aside from an attenuation of the estimates for black HCWs (vs white) in outcome 3 (online supplemental table 9).

## Discussion

This analysis of a large, ethnically diverse cohort of NHS HCWs has several novel findings. Overall, four in five NHS HCWs reported being offered a COVID-19 risk assessment, and among those offered an assessment, 9 in 10 reported completing one. Ethnic minority groups were more likely to be offered a risk assessment and to complete one once offered compared with white groups. Among those who completed a risk assessment (and did not indicate workplace adjustments were unnecessary), 7 in 10 reported having work adjustments made, but this was less likely for ethnic minority HCWs. Finally, in those whose work did not change (and who and did not indicate workplace adjustments were unnecessary), half (49.2%) reported unfulfilled wishes for workplace adjustments, and this was more likely among HCWs from black and Asian ethnic groups than white groups.

Our findings indicate that ethnicity was being recognised as a risk factor for adverse outcomes from COVID-19 by both NHS employers (evidenced by the higher odds of being offered a risk assessment in ethnic minority groups compared with white groups) and by individual HCWs (evidenced by the higher odds of completing an assessment once offered in ethnic minority groups compared with white groups). While ethnicity was proven a major factor in mediating the risk of SARS-CoV-2 infection in HCWs^1 7 23^ and, therefore, represents an important criterion to include in COVID-19 risk assessments, it is important to note that targeting risk assessments at those from ethnic minority groups has the potential to create stigma;^24 25^ thus, adopting the recommended approach of a universal risk assessment that takes account of ethnicity may be preferable.^16^

Given that the NHS did recognise that risk assessments should consider ethnicity,^15 26^ it is surprising that ethnic minority HCWs were less likely to have adjustments made (after exclusion of those who reported work adjustments were unnecessary). This is driven both by the increased proportion of ethnic minority HCWs (compared with white HCWs) reporting not wanting changes made and those reporting wanting changes made and not getting them. Previous work has suggested that clinicians from ethnic minority groups experience a dilemma of choosing between their clinical and leadership responsibilities and risks to their own health and that of their loved ones from COVID-19.^24 27^ Such dilemmas may also partly explain our finding that those who had physical contact with patients with COVID-19 were less likely to have work changed after a risk assessment than those who had no (or remote) contact only. Additionally, HCWs may fear barriers to career progression that could follow redeployment or other workplace amendments and such concerns may not affect different ethnic groups equally.^13^ They may also fear being judged negatively by colleagues.^24^ Race discordance between managers and staff may make conversations around risk assessment more difficult.^24^ It should also be noted that there has been criticism of the lack of consistency across the NHS in the risk assessment process^13^ with experiences ranging from informal conversations with managers to meetings with formal documentation.^24^ Therefore, it is possible that not all risk assessments took ethnicity into account.

Explanations for the increased likelihood for ethnic minority staff to indicate that they wanted changes to be made but did not happen may relate to structural discrimination. It has been suggested that ethnic minority HCWs feel less empowered to ask for risk assessments,^13^ which in itself may be related to factors such as a lack of trust in their employing organisation^21^ or due to their experiences of harassment or bullying at work.^28^ These same factors may also influence not feeling empowered to ask for changes to working practices from employers/managers. Specific occupational characteristics such as seniority within a healthcare team (a factor we show to be important in influencing decisions around risk assessment) or healthcare specialty may also impact upon risk assessment outcome. Ethnic minority HCWs are more likely to work in junior positions,^28^ which may involve greater patient contact. It may, therefore, be more difficult for employers/managers to usefully redeploy these HCWs into different roles or make amendments to their level of contact with patients when compared with a more senior HCW who could take on greater administrative responsibilities at the expense of patient contact. This may also explain why staff in administrative roles are more likely to have workplace adjustments made than medical staff. Establishing the groups of HCWs who had unfulfilled wishes for workplace adjustment is of critical importance. HCWs continuing to work in an environment where they feel at risk is likely to have a detrimental effect on well-being and undermine trust in employing organisation (a factor shown to relate to other ethnic inequalities in HCWs such as vaccine hesitancy).^29^

General practitioners and those in dental roles had lower odds of being offered risk assessments than consultants or medical staff, respectively. This may be due to evidence suggesting that community HCWs are at lower risk of COVID-19^5^ than hospital staff, which might lead to the perception that risk assessment was less important in these groups. Community HCWs may also have less access to occupational health services than their counterparts in acute trusts, which could account for these differences.^30^

Previous COVID-19 was associated with lower odds of being offered and having adjustments made as a result of risk assessment. SARS-CoV-2 infection provides immune protection against reinfection and thus might represent an important factor to be taken account of during risk assessment. However, this protection wanes and may be evaded by new SARS-CoV-2 variants.^31 32^ It would be important to reassess such people in the light of current knowledge, particularly considering the risk of morbidity from long COVID.

Our work has several limitations: we are unable to determine the exact outcome of individual risk assessments. We have assumed that staff assessed as being at low risk by whichever risk assessment tool was employed would respond to the question about whether workplace changes were made with ‘No, because it did not need to’. It is possible, however, that such HCWs could respond with ‘No, but I wanted it to’ if the risk assessment outcome was discordant with the HCWs’ perceived level of risk. We are also unable to determine whether changes made to working practices were appropriate and acceptable to the HCWs.

Selection bias may have affected our results. HCWs who responded to our survey may also be more likely to respond to an offer of a COVID-19 risk assessment; therefore, we may have overestimated the proportion of HCWs who completed an assessment once offered. As we administered the questionnaire in December 2020 and ask about occupational circumstances at the time of the first UK lockdown in March 2020, we may have introduced recall bias. The cross-sectional nature of the study means we cannot be definitive about the direction of any association; however, in planning our analysis, we were careful to omit variables that may have been particularly affected by reverse causality from the models. We did not ask participants for the date of their risk assessment. We have, therefore, used variables concerning SARS-CoV-2 infection and exposure that we can be sure predate the roll-out of NHS COVID-19 risk assessments; however, these may not accurately reflect the occupational circumstances of the HCWs at the time of risk assessment. Through exclusion of those not working during lockdown and at the time of questionnaire response, we will have excluded some HCWs who were ‘shielding’ (ie, avoiding contact with others as a means of protecting themselves against infection). These HCWs are likely to have had changes made to their working practices as they will be among the most vulnerable to severe COVID-19; therefore, this could lead to underestimating the proportion of HCWs who had adjustments made to working practices. Furthermore, it is possible that risk assessment (whether formal or informal) occurred at some point prior to the first UK national lockdown; therefore, in some cases, our occupational COVID-19 exposure variable may result in occupational circumstances after risk assessment. We do not account for clustering by NHS Trust; authors do not have access to the specific NHS Trusts at which participants work to protect confidentiality. Previous work in this cohort which stratified respondents by region of workplace^5 18 33^ did not indicate that any particular region was dominant; thus, we do not anticipate that this had a major impact on results.

Universal risk assessments, which are repeated to take account of changing risk factors, such as vaccination, new variants of SARS-CoV-2, changing job roles and personal protective equipment access, are critical to protect HCWs against SARS-CoV-2 infection and its sequelae. We have determined that a large proportion of NHS staff have completed a COVID-19 risk assessment and that there are ethnic differences in NHS COVID-19 risk assessment outcomes. While it is encouraging that employers seem to have taken account of the increased risk of infection and severe outcomes from COVID-19 faced by ethnic minority HCWs when offering risk assessments, we caution that the likelihood of workplace adjustments being made after risk assessment may be lower in those from ethnic minority groups than white groups. These findings are concerning and warrant further research in a larger, unselected cohort.

## Data Availability

Data are available upon reasonable request. To access data or samples produced by the UK-REACH Study, the working group representative must first submit a request to the Core Management Group by contacting the UK-REACH Project Manager in the first instance. For ancillary studies outside of the core deliverables, the Steering Committee will make final decisions once they have been approved by the Core Management Group. Decisions on granting the access to data/materials will be made within 8 weeks. Third-party requests from outside the project will require explicit approval of the Steering Committee once approved by the Core Management Group. Note that should there be significant numbers of requests to access data and/or samples, then a separate Data Access Committee will be convened to appraise requests in the first instance.
