# Supplementary material for: Coverage, completion and outcomes of COVID-19 risk assessments in a multi-ethnic nationwide cohort of UK healthcare workers: a cross-sectional analysis from the UK-REACH Study
Source: Occup Environ Med. 2023 May 23;80(7):399–406. doi: 10.1136/oemed-2022-108700 (PMC10314065; doi:10.1136/oemed-2022-108700)
Supplement: Supplementary data [file oemed-2022-108700supp001.pdf]

Supplementary information

Coverage, completion and outcomes of COVID-19 risk assessments in a multi-ethnic nationwide cohort of UK healthcare workers: a cross sectional analysis from the UK-REACH study

Christopher A. Martin[1,2], Katherine Woolf[3], Luke Bryant[1], Charles Goss[4], Mayuri Gogoi[1], Susie Lagrata[5], Padmasayee Papineni[6], Irtiza Qureshi[7], Fatimah Wobi[8,9], Laura B. Nellums[7], Kamlesh Khunti[10], Manish Pareek[1,2]

On behalf of the UK-REACH Study Collaborative Group+

Corresponding author mp426@le.ac.uk

+Manish Pareek (Chief investigator), Laura Gray (University of Leicester), Laura Nellums (University of Nottingham), Anna L Guyatt (University of Leicester), Catherine John (University of Leicester), I Chris McManus (University College London), Katherine Woolf (University College London), Ibrahim Abubakar (University College London), Amit Gupta (Oxford University Hospitals), Keith R Abrams (University of York), Martin D Tobin (University of Leicester), Louise Wain (University of Leicester), Sue Carr (University Hospital Leicester), Edward Dove (University of Edinburgh), Kamlesh Khunti (University of Leicester), David Ford (University of Swansea), Robert Free (University of Leicester).

1. Department of Respiratory Sciences, University of Leicester, Leicester, UK

2. Department of Infection and HIV Medicine, University Hospitals of Leicester NHS Trust, Leicester, UK

3. University College London Medical School, London, UK

4. Department of Occupational Health, University Hospitals of Leicester NHS Trust

5. University College London Hospitals NHS Foundation Trust, Leicester, UK

6. Department of Infectious Diseases, Ealing Hospital, London North West University Healthcare NHS Trust, London

7. Population and Lifespan Sciences, School of Medicine, University of Nottingham, Nottingham, UK

8. Public Health Institute, Liverpool John Moores University, Liverpool, UK

9. School of Law, University of Leicester, Leicester, UK

10. Diabetes Research Centre, University of Leicester, Leicester, UK

Supplementary information

Supplementary Text 1. UK-REACH – overview and recruitment

Overview

UK-REACH is a research programme consisting of multiple sub-studies. The overarching aim of the programme is to determine whether, and to what degree, there has been a disproportionate impact of the COVID-19 pandemic on ethnic minority HCWs.

This work uses data from the baseline questionnaire of the nationwide cohort study, which was administered between December 2020 and March 2021. Details of the design, sampling and measures included in the questionnaire can be found in the study protocol[15], the cohort profile[16], the data dictionary (https://www.uk-reach.org/data-dictionary), and in other published work[6 17-19].

Recruitment

We recruited individuals aged 16 years or over, living in the UK and employed as HCWs or ancillary workers in a healthcare setting and/or registered with one of seven major UK professional regulatory bodies:

- The General Medical Council (GMC)
- The Nursing and Midwifery Council (NMC)
- The General Dental Council (GDC)
- The Health and Care Professions Council (HCPC)
- The General Optical Council (GOC)
- The General Pharmaceutical Council (GPC)
- The Pharmaceutical Society of Northern Ireland (PSNI)

Professional regulators distributed emails to their registrants embedded with a hyperlink to the study website. Those interested could create a user profile, read the participant information sheet and, provided they were willing, sign an online consent form. Participants were then asked to complete the online questionnaire. The sample was supplemented by recruitment of eligible HCWs through

BMJ Publishing Group Limited (BMJ) disclaims all liability and responsibility arising from any reliance placed on this supplemental material which has been supplied by the author(s)

Supplementary information

participating healthcare Trusts, and advertising on social media and in newsletters. These participants were also provided with a link to the study website and followed the same procedure as above.

Participation rates at each stage are reported as recommended by the Checklist for Reporting Results of Internet E-Surveys (CHERRIES).

We recruited individuals aged 16 years or over, living in the UK and employed as HCWs or ancillary workers in a healthcare setting and/or registered with one of seven major UK professional regulatory bodies.

Professional regulators distributed emails to their registrants embedded with a hyperlink to the study website. Those interested could create a user profile, read the participant information sheet and, provided they were willing, sign an online consent form. Participants were then asked to complete the online questionnaire. The sample was supplemented by recruitment of eligible HCWs through participating healthcare Trusts, and advertising on social media and in newsletters. These participants were also provided with a link to the study website and followed the same procedure as above.

Participation rates at each stage are reported as recommended by the Checklist for Reporting Results of Internet E-Surveys (CHERRIES).

Supplementary Text 2. Involvement and engagement

We worked closely with a Professional Expert Panel of HCWs from a range of ethnic backgrounds, healthcare occupations, and sexes, as well as with national and local organisations (see study protocol).[20] The panel were involved in the design of the survey instruments and assisted in developing the research question and analysis plan. Some members have critically reviewed the manuscript and are co-authors on the study.

Supplementary information

Supplementary Text 3. Description of the cohort by responses to questionnaire items

Responses to questionnaire item 1 - "Have you been offered an NHS COVID-19 risk assessment at work?"

6,575 (76.0%) of HCWs reported that they had been offered a risk assessment and chose to complete it; 508 (5.9%) reported being offered a risk assessment but choosing not to complete it; 1,022 (11.8%) reported not being offered a risk assessment and 544 (6.3%) reported having not heard of COVID-19 risk assessments (Supplementary Table 2).

The proportion of those who reported not having heard of COVID-19 risk assessments was lower in Asian and Black ethnic groups when compared to the proportion of the total cohort from these ethnic groups (Asian 11.6% vs 21.0%, Black 1.5% vs 4.3%). A similar pattern was seen for reporting not being offered a risk assessment (Asian 12.6% vs 21.0%, Black 2.1% vs 4.3%) and for being offered a risk assessment but not completing it (Asian 14.6% vs 21.0%, Black 2.8% vs 4.3%).

Compared to the cohort as a whole, the proportion of those reporting not having heard of COVID-19 risk assessments who were working in medical roles were lower (21.8% vs 28.3%) and allied health professional roles were higher (48.5% vs 40.2%). The proportion of those who were offered a risk assessment and chose not to complete it who worked in dental roles was lower than the proportion of dentists in the cohort (1.4% vs 3.3%).

A higher proportion of HCWs who reported being offered but not completing a risk assessment and not having heard of risk assessments had no long-term physical health conditions (83.1% and 83.4 %) when compared to the whole cohort (73.1%).

Median score on the perceived risk of COVID-19 hospitalisation scale in the group who reported being offered but not completing a risk assessment was half that of the cohort overall (10 IQR 5 – 30 vs 20 IQR 5 – 50).

Responses to questionnaire item 2 - "Did your work change as a result of the NHS COVID-19 risk assessment result?"

Supplementary information

6,543 HCWs who reported having completed a risk assessment provided information on whether their work changed as a result. 4,579 (70.0%) reported their work did not change because it did not need to; 294 (4.5%) reported that their work did not change because they didn't want it to; 285 (4.4%) reported that their work didn't change but they wanted it to; 1,385 (21.2%) reported that their work had changed in some way (Supplementary Table 3).

When compared to the total proportions of those who completed a risk assessment, a higher proportion of the HCWs who reported that their work did not change as a result of the risk assessment because they did not want it to were from ethnic minority groups (Asian 35.0% vs 23.6%; Black 6.1% vs 5.0%; Mixed 9.2% vs 4.5%; Other 4.1% vs 2.3%). A similar pattern was seen amongst those who reported that their work didn't change but they wanted it to (Asian 40.4% vs 23.6%; Black 9.1% vs 5.0%; Mixed 7.4% vs 4.5%; Other 3.2% vs 2.3%).

There were a smaller proportion of female HCWs in the group reporting that their work did not change because they didn't want it to when compared to the total cohort who reported completing a risk assessment (62.5% vs 74.9%).

A higher proportion of those reporting that their work did not change because they did not want it to were working in medical roles than the proportion of those in medical roles within the whole cohort who completed a risk assessment (43.1% vs 29.0%). Nurses made up a larger proportion of the group reporting that their work did not change despite wanting it to (27.0% vs 22.7%).

When comparing the group of HCWs who reported that their work did not change because they didn't want it to with the cohort who completed a risk assessment, a much high proportion were made up of those who reported physical contact with COVID-19 patients during lockdown (61.2% vs 44.2%) the same group made up a smaller proportion of the group who reported having changes made to working practices after risk assessment (29.7% vs 44.2%).

Supplementary information

Supplementary Table 1. Derivation of variables used in the analysis

Variable	Description
**Ethnicity**	Categorical variable. Participants were asked to select their ethnicity from a list of the 18 Office for National Statistics categories:<br>Asian/Asian British – Indian<br>Asian/Asian British – Pakistani<br>Asian/Asian British – Bangladeshi<br>Asian/Asian British – Chinese<br>Asian/Asian British - Any other Asian background<br>Black/African/Caribbean/Black British - African<br>Black/African/Caribbean/Black British – Caribbean<br>Black/African/Caribbean/Black British - Any other Black/African/Caribbean background<br>Mixed/Multiple ethnic groups - White and Black Caribbean<br>Mixed/Multiple ethnic groups - White and Black African<br>Mixed/Multiple ethnic groups - White and Asian<br>Mixed/Multiple ethnic groups - Any other Mixed/multiple ethnic background<br>White - English/Welsh/Scottish/Northern Irish/British<br>White – Irish<br>White - Gypsy or Irish Traveller<br>White - Any other white background<br>Other ethnic group – Arab<br>Other ethnic group - Any other ethnic background<br><br>These were categorised into the 5 broader Office for National Statistics ethnicity categories (Asian, Black, Mixed, White, Other).
**Age**	Continuous variable. Age in years. Derived from date of birth entered by participants at registration.
**Sex**	Binary variable. Participants were asked their sex assigned at birth.
**Migration status**	Binary variable - Born in the UK vs Born overseas. Participants were asked whether they were born in the UK.
**Occupation**	Categorical variable. Participants were asked to select their main job/role. Categorised as below:<br><br>**Doctor or medical support** - Doctor, Advanced Critical Care Practitioner, Anaesthesia associate, Surgical Care Practitioner, Other medical associate<br><br>**Nurse, NA or Midwife -** Advanced Nurse Practitioner, Healthcare assistant, Maternity support worker, Midwife, Nurse, Nursing Associate, Other nursing and midwifery role,<br><br>**Allied Health Professional (including pharmacists, ambulance workers and those in optical roles)** - Arts therapist, Biomedical scientist, Chiropodist/Podiatrist, Clinical scientist, Dietician, Hearing aid dispenser, Occupational therapist, Operating department practitioner, Orthoptist, Physiotherapist, Practitioner psychologist, Prosthetist / Orthotist, Radiographer, Speech and language therapist, Other Allied Health Professional role, Emergency medical , Paramedic , Other ambulance role, OT Support , Phlebotomist, Physiotherapy Assistant, Radiography Other clinical support role , Pharmacist , Pharmacy technician, Other pharmacy role, Optical - Dispensing optician, Optometrist, Other Optical role<br><br>**Dental -** Clinical dental technician, Dental Hygienist, Dental nurse, Dental technician, Dentist, Other dental role<br><br>**Admin, estates or other –** Administration, Catering services, Domestic services, Estates services, Porter, Other
**Level of exposure to COVID-19 patients during lockdown.**	Categorical variable (ordered). Participants were asked how many COVID-19 patients they attended to on a weekly basis during lockdown. There were three separate questions relating to how many patients they saw i) remotely; ii) face-to-face with social distancing; iii) with physical contact. These questions were used to derive a variable which indicates the 'closest contact' the participant indicated they had with COVID-19 patients i.e. no contact (or remote contact only), face-to-face with social distancing but no physical contact or physical contact.
**Number of long-term physical health conditions.**	Categorical variable (ordered). Number of long-term physical health conditions from a selection of diabetes, hypertension, heart disease, asthma, lung disease (other than asthma), kidney or liver disease, neurological disease, cancer, immunosuppression or organ transplant (categorised as 0, 1 or ≥2).

Supplementary information

**Body mass index**	Categorical variable (ordered). Participants were asked to enter their height and weight. This was used to derive body mass index in kg/m^2. This was categorised using recognised cut-off values into <25, 25 – 30 and ≥30.
**Previous SARS-CoV-2 infection status.**	Binary variable. Uninfected vs infected. Self-reported confirmed or suspected SARS-CoV-2 infection status on 1st May 2020 (categorised as uninfected and infected). As we do not have information on date of risk assessment, we selected this threshold date to balance the likelihood that the infection event occurred before a risk assessment took place and to capture enough infection events to determine the association between infection status and our outcome measures.
**Perceived risk of hospitalisation with COVID-19 after infection with SARS-CoV-2**	Continuous variable. Participants were asked " If you do catch coronavirus, what do you think are¬†your¬†chances of needing hospital treatment? Please enter a value on a scale from 0 to 100, where 0 means there is no possibility that you will and 100 means that you definitely will."
**Perceived risk of unknowingly spreading COVID-19**	Categorical variable (ordered). Participants were asked " How concerned are you that you might unknowingly spread COVID-19 to others?" and could answer on the following scale " 1, Not at all concerned | 2, A little concerned | 3, Quite concerned | 4, Very concerned | 99, Prefer not to answer"
**Cohabitation with those over 65 years old**	Binary variable. Either does or does not live with someone over the age of 65 years. Participants were asked to give the ages of each member of their household.

For further information on questionnaire variables, refer to the UK-REACH data dictionary (https://www.uk-reach.org/data-dictionary).

Supplementary information

Supplementary Table 2. Description of the cohort stratified by response to the questionnaire item "Have you been offered an NHS COVID-19 risk assessment at work?"

Variable	Total<br>N=8649	Not heard of COVID-19 risk assessments<br>N=544	Not been offered a COVID-19 risk assessment<br>N=1,022	Offered a COVID-19 risk assessment but chose not to complete it<br>N=508	Offered a COVID-19 risk assessment and completed it<br>N=6,575
**Ethnicity**					
White	5,900 (68.2)	437 (80.3)	833 (81.5)	385 (75.8)	4,245 (64.6)
Asian	1,820 (21.0)	63 (11.6)	129 (12.6)	74 (14.6)	1,554 (23.6)
Black	371 (4.3)	8 (1.5)	21 (2.1)	14 (2.8)	328 (5.0)
Mixed	360 (4.2)	20 (3.7)	24 (2.4)	22 (4.3)	294 (4.5)
Other	198 (2.3)	16 (2.9)	15 (1.5)	13 (2.6)	154 (2.3)
**Age in years, med (IQR)**	44 (34-53)	43 (33 – 53)	45 (35 – 54)	43 (33 – 52)	44 (34 – 53)
**Sex**					
Male	2,202 (25.5)	166 (30.7)	238 (23.3)	150 (29.6)	1,648 (25.1)
Female	6,429 (74.5)	374 (69.3)	783 (76.7)	356 (70.4)	4,916 (74.9)
**Occupation**					
Medical / medical support	2,366 (28.3)	114 (21.8)	254 (26.0)	148 (30.0)	1,850 (29.1)
Nursing (inc. midwives, nursing associates)	1,903 (22.8)	107 (20.4)	243 (24.9)	107 (21.7)	1,446 (22.7)
Allied health professionals*	3,360 (40.2)	254 (48.5)	388 (39.7)	199 (40.4)	2,519 (39.6)
Dental	289 (3.5)	29 (5.5)	50 (5.1)	7 (1.4)	203 (3.2)
Administrative / estates / other	446 (5.3)	20 (3.8)	43 (4.4)	32 (6.5)	351 (5.5)
**Migration status**					
Born in the UK	6,249 (72.4)	422 (77.6)	835 (81.9)	393 (77.8)	4,599 (70.1)
Born overseas	2,379 (27.6)	122 (22.4)	185 (18.1)	112 (22.2)	1,960 (29.9)
**Exposure to COVID-19 patients during lockdown**					
None (or remote contact only)	4,122 (48.0)	226 (41.7)	459 (45.5)	204 (40.5)	3,233 (49.6)
Face-to-face with social distancing only	520 (6.1)	22 (4.1)	51 (5.1)	42 (8.3)	405 (6.2)
Physical contact	3,938 (45.9)	294 (54.2)	500 (49.5)	258 (51.2)	2,886 (44.2)
**Number of long-term physical health conditions†**					
0	5,819 (73.1)	407 (83.4)	713 (76.3)	379 (83.1)	4,320 (71.0)
1	1,721 (21.6)	67 (13.7)	189 (20.2)	61 (13.4)	1,404 (23.1)
≥2	419 (5.3)	14 (2.9)	32 (3.4)	16 (3.5)	357 (5.9)
**Body mass index (kg/m^2)**					
< 25	3,675 (48.7)	212 (45.5)	430 (49.2)	210 (49.9)	2,823 (48.8)
≥ 25 & < 30	2,360 (31.3)	149 (32.0)	276 (31.6)	132 (31.4)	1,803 (31.2)

Supplementary information

≥30	1,513 (20.1)	105 (22.5)	168 (19.2)	79 (18.8)	1,161 (20.1)
**Perceived risk of being hospitalised with COVID-19 in the next 6 months (scale 0 – 100), med(IQR)**	20 (5 – 50)	15 (5 – 50)	10 (5 – 40)	10 (5 – 30)	20 (5 – 50)
**Perceived likelihood of unknowingly spreading COVID-19**					
Not at all likely	1,071 (13.1)	63 (12.3)	112 (11.6)	71 (14.7)	825 (13.3)
A little likely	3,023 (37.0)	187 (36.5)	351 (36.3)	181 (37.5)	2,304 (37.0)
Quite likely	2,197 (26.9)	132 (25.8)	274 (28.4)	133 (27.5)	1,658 (26.7)
Very likely	1,891 (23.1)	130 (25.4)	229 (23.7)	98 (20.3)	1,434 (23.1)
**SARS-CoV-2 infection status (at 1st May 2020)**					
Uninfected	7,075 (86.9)	435 (85.0)	807 (84.4)	404 (84.0)	5,429 (87.7)
Infected	1,068 (13.1)	77 (15.0)	149 (15.6)	77 (16.0)	765 (12.4)
**Cohabitation with those over 65 years old**					
Does not live with someone over the age of 65	7,842 (93.0)	496 (93.8)	939 (94.4)	457 (93.5)	5,950 (92.7)
Lives with someone over the age of 65	591 (7.0)	33 (6.2)	56 (5.6)	32 (6.5)	470 (7.3)

Supplementary Table 2 provides a description of the cohort stratified by their answer to the question “Have you been offered an NHS COVID-19 risk assessment at work?”. All data are n(%) unless stated otherwise.

*Includes pharmacists, health scientists, ambulance workers and those in optical roles. †Includes diabetes, heart disease, hypertension, previous stroke, kidney or liver disease, asthma, lung condition other than asthma, cancer, neurological disease, organ transplant and immunosuppression.

Supplementary information

Supplementary Table 3. Description of those who completed an NHS COVID-19 risk assessment stratified by their response to the question "Did your work change as a result of the NHS COVID-19 risk assessment result?"

Variable	Total N=6,543	No, because it didn't need to N=4,579	No, because I didn't want it to N=294	No, but I did want it to N=285	Yes N=1,385
**Ethnicity**					
White	4,230 (64.7)	3,118 (68.1)	134 (45.6)	114 (40.0)	864 (62.4)
Asian	1,545 (23.6)	990 (21.6)	103 (35.0)	115 (40.4)	337 (24.3)
Black	324 (5.0)	198 (4.3)	18 (6.1)	26 (9.1)	82 (5.9)
Mixed	294 (4.5)	171 (3.7)	27 (9.2)	21 (7.4)	75 (5.4)
Other	150 (2.3)	102 (2.2)	12 (4.1)	9 (3.2)	27 (2.0)
**Age in years, med (IQR)**	44 (34 – 53)	43 (33 – 52)	46 (36 – 54)	43 (33 – 54)	49 (39 – 56)
**Sex**					
Male	1,638 (25.1)	1,116 (24.4)	110 (37.5)	74 (26.1)	338 (24.5)
Female	4,894 (74.9)	3,459 (75.6)	183 (62.5)	210 (73.9)	1,042 (75.5)
**Occupation**					
Medical / medical support	1,840 (29.0)	1,336 (30.0)	124 (43.1)	87 (31.8)	293 (22.1)
Nursing (inc. midwives, nursing associates)	1,441 (22.7)	948 (21.3)	56 (19.4)	74 (27.0)	363 (27.3)
Allied health professionals*	2,509 (39.6)	1,776 (39.9)	96 (33.3)	93 (33.9)	544 (40.9)
Dental	203 (3.2)	132 (3.0)	6 (2.1)	12 (4.4)	53 (4.0)
Administrative / estates / other	349 (5.5)	259 (5.8)	6 (2.1)	8 (2.9)	76 (5.7)
**Migration status**					
Born in the UK	4,586 (70.3)	3,307 (72.4)	174 (59.2)	170 (59.7)	935 (67.8)
Born overseas	1,941 (29.7)	1,262 (27.6)	120 (40.8)	115 (40.4)	444 (32.2)
**Exposure to COVID-19 patients during lockdown**					
None (or remote contact only)	3,221 (49.6)	2,128 (46.8)	89 (30.6)	125 (44.0)	879 (64.2)
Face-to-face with social distancing only	402 (6.2)	279 (6.1)	24 (8.3)	15 (5.3)	84 (6.1)
Physical contact	2,869 (44.2)	2,141 (47.1)	178 (61.2)	144 (50.7)	406 (29.7)
**Number of long-term physical health conditions†**					
0	4,299 (71.1)	3,440 (81.2)	129 (47.1)	146 (57.5)	584 (45.5)
1	1,396 (23.1)	710 (16.8)	116 (42.3)	84 (33.1)	486 (37.8)
≥2	355 (5.9)	87 (2.1)	29 (10.6)	24 (9.5)	215 (16.7)
**Body mass index (kg/m^2)**					
< 25	2,809 (48.8)	2,129 (52.5)	113 (42.2)	97 (40.3)	470 (39.3)
≥ 25 & < 30	1,795 (31.2)	1,235 (30.5)	84 (31.3)	88 (36.5)	388 (32.5)

Supplementary information

≥30	1,153 (20.0)	689 (17.0)	71 (26.5)	56 (23.2)	337 (28.2)
**Perceived risk of being hospitalised with COVID-19 in the next 6 months (scale 0 – 100), med(IQR)**	20 (5 – 50)	10 (5 – 30)	20 (10 – 50)	35 (10 – 52.5)	30 (10 – 50)
**Perceived likelihood of unknowingly spreading COVID-19**					
Not at all likely	822 (13.3)	535 (12.3)	47 (16.9)	32 (12.3)	208 (16.1)
A little likely	2,292 (37.0)	1,701 (39.1)	94 (33.7)	75 (28.7)	422 (32.6)
Quite likely	1,651 (26.7)	1,200 (27.5)	67 (24.0)	66 (25.3)	318 (24.5)
Very likely	1,426 (23.0)	919 (21.1)	71 (25.5)	88 (33.7)	348 (26.9)
**SARS-CoV-2 infection status (at 1st May 2020)**					
Uninfected	5,403 (87.7)	3,777 (87.2)	238 (84.4)	222 (85.7)	1,166 (90.0)
Infected	760 (12.3)	554 (12.8)	40 (15.6)	37 (14.3)	129 (10.0)
**Cohabitation with those over 65 years old**					
Does not live with someone over the age of 65	5,923 (92.7)	4,203 (93.9)	265 (91.4)	248 (89.5)	1,207 (89.6)
Lives with someone over the age of 65	466 (7.3)	272 (6.1)	25 (8.6)	29 (10.5)	140 (10.39)

Supplementary Table 3 provides a description of the cohort stratified by their answer to the question “Have you been offered an NHS COVID-19 risk assessment at work?”. All data are n(%) unless stated otherwise.

*Includes pharmacists, health scientists, ambulance workers and those in optical roles. †Includes diabetes, heart disease, hypertension, previous stroke, kidney or liver disease, asthma, lung condition other than asthma, cancer, neurological disease, organ transplant and immunosuppression

Supplementary information

Supplementary Table 4. Logistic regression table for outcomes 1 and 2 to show numerical adjusted odds ratios and p values (data displayed graphically in Figure 2).

	OUTCOME 1 – OFFERED A RISK ASSESSMENT (n=8,649)		OUTCOME 2 – COMPLETED A RISK ASSESSMENT (n=7,083)	
	aOR (95%CI)	P value	aOR (95%CI)	P value
**Ethnicity**				
White	Ref	-	Ref	-
Asian	2.37 (1.95 – 2.87)	<0.001	2.17 (1.60 – 2.94)	<0.001
Black	3.11 (2.09 – 4.62)	<0.001	2.27 (1.28 – 4.00)	0.005
Mixed	2.03 (1.46 – 2.81)	<0.001	1.38 (0.88 – 2.18)	0.16
Other	1.47 (0.97 – 2.22)	0.07	1.24 (0.67 – 2.29)	0.50
**Age,** per decade increase	1.02 (0.98 – 1.08)	0.34	1.14 (1.05 – 1.23)	0.002
**Sex**				
Male	Ref	-	Ref	-
Female	1.20 (1.05 – 1.38)	0.007	1.41 (1.14 – 1.75)	0.002
**Occupation***				
Medical	Ref	-	Ref	-
Nursing	1.15 (0.96 – 1.38)	0.12	1.34 (1.01 – 1.79)	0.046
Allied Health Professional	1.07 (0.91 – 1.26)	0.40	1.29 (1.01 – 1.66)	0.04
Dental	0.60 (0.45 – 0.80)	<0.001	2.83 (1.30 – 6.17)	0.009
Admin/estates/other	1.59 (1.18 – 2.14)	0.002	1.17 (0.77 – 1.77)	0.46
**Migration status**				
Born in the UK	Ref	-	Ref	-
Born overseas	1.20 (1.02 – 1.41)	0.03	1.20 (0.92 – 1.55)	0.17
**Exposure to COVID-19 patients**				
None (or remote only)	Ref	-	Ref	-
Face-to-face with social distancing	1.12 (0.86 – 1.45)	0.41	0.60 (0.42 – 0.85)	0.004
Physical contact	0.70 (0.62 – 0.79)	<0.001	0.72 (0.58 – 0.88)	0.002
**Number of long-term physical health conditions**				
0	Ref	-	Ref	-
1	1.38 (1.19 – 1.60)	<0.001	1.89 (1.43 – 2.50)	<0.001
≥2	1.93 (1.42 – 2.64)	<0.001	1.99 (1.19 – 3.32)	0.009
**BMI (kg/m^2)**				
< 25	Ref	-	Ref	-
≥ 25 to < 30	0.96 (0.84 – 1.10)	0.53	1.00 (0.80 – 1.26)	0.99
≥ 30	1.00 (0.85 – 1.17)	1.00	1.11 (0.84 – 1.46)	0.47
**SARS-CoV-2 infection status**				
Uninfected	Ref	-	Ref	-
Infected	0.81 (0.69 – 0.95)	0.01	0.78 (0.60 – 1.02)	0.07
**Perceived risk of hospitalisation due to COVID-19** (scale 0 – 100), per 10 point increase	-	-	1.08 (1.04 – 1.13)	0.001
**Perceived risk of spreading COVID-19**				
Not at all likely	-	-	Ref	-
A little likely			1.16 (0.86 – 1.55)	0.33
Quite likely			1.20 (0.88 – 1.63)	0.26
Very likely			1.31 (0.94 – 1.83)	0.11
**Cohabitation**				
Does not live with someone over 65 years old	-	-	Ref	-
Lives with someone over 65 years old			0.94 (0.64 – 1.38)	0.21

Supplementary Table 6 shows the results of multivariable logistic regression as adjusted odds ratios, 95% confidence intervals and p values for the association of sociodemographic, occupational and perceived risk variables with outcome 1 (offered a risk assessment) and outcome 2 (completed a risk assessment once offered). Odds ratios are adjusted for age, sex, ethnicity, occupation and migration status. We did not include the 'perceived risk' variables in the analysis of outcome 1 as we felt these would not have influenced whether a HCW was offered a risk assessment.

*In the occupation variable, nursing includes midwives, nursing associates and healthcare assistants; allied health professionals includes pharmacists, healthcare scientists, ambulance workers and those in optical roles.

BMI - Body mass index; COVID-19 - coronavirus disease 2019; SARS-CoV-2 - Severe acute respiratory syndrome coronavirus-2

Supplementary information

Supplementary Table 5. Logistic regression table for outcomes 3 and 4 to show numerical adjusted odds ratios and p values (data displayed graphically in Figure 2).

	**OUTCOME 3 – WORKING PRACTICES CHANGED (n=1,964)**		**OUTCOME 4 – WANTED CHANGES BUT WORK DIDN'T CHANGE (n=442)**	
	**aOR (95%CI)**	**P value**	**aOR (95%CI)**	**P value**
**Ethnicity**				
White	Ref	-	Ref	-
Asian	0.55 (0.41 – 0.73)	<0.001	2.18 (1.33 – 3.59)	0.002
Black	0.60 (0.39 – 0.92)	0.02	2.15 (1.05 – 4.42)	0.04
Mixed	0.51 (0.34 – 0.76)	0.001	1.12 (0.58 – 2.16)	0.73
Other	0.45 (0.24 – 0.86)	0.02	1.48 (0.55 – 4.03)	0.44
**Age,** per decade increase	1.31 (1.19 – 1.43)	<0.001	0.89 (0.77 – 1.04)	0.14
**Sex**				
Male	Ref	-	Ref	-
Female	1.28 (1.00 – 1.63)	0.048	1.59 (1.08 – 2.34)	0.02
**Occupation**				
Medical	Ref	-	Ref	-
Nursing	1.36 (0.99 – 1.87)	0.06	2.39 (1.41 – 4.03)	0.001
Allied Health Professional*	1.72 (1.31 – 2.27)	<0.001	1.61 (1.03 – 2.52)	0.04
Dental	1.70 (0.94 – 3.05)	0.08	3.07 (1.07 – 8.79)	0.04
Admin/estates/other	3.04 (1.63 – 5.66)	<0.001	2.47 (0.79 – 7.78)	0.44
**Migration status**				
Born in the UK	Ref	-	Ref	-
Born overseas	1.17 (0.90 – 1.52)	0.23	0.92 (0.60 – 1.41)	0.70
**Exposure to COVID-19 patients**				
None (or remote only)	Ref	-	Ref	-
Face-to-face with social distancing	0.52 (0.34 – 0.79)	0.002	0.50 (0.24 – 1.03)	0.06
Physical contact	0.38 (0.31 – 0.48)	<0.001	0.58 (0.39 – 0.85)	0.006
**Number of long-term physical health conditions**				
0	Ref	-	Ref	-
1	1.08 (0.86 – 1.35)	0.53	0.71 (0.47 – 1.06)	0.10
≥2	1.73 (1.23 – 2.45)	0.002	0.87 (0.45 – 1.68)	0.68
**BMI (kg/m^2)**				
< 25	Ref	-	Ref	-
≥ 25 to < 30	1.00 (0.77 – 1.30)	0.98	1.22 (0.79 – 1.88)	0.38
≥ 30	0.98 (0.74 – 1.29)	0.89	1.08 (0.67 – 1.75)	0.75
**SARS-CoV-2 infection status**				
Uninfected	Ref	-	Ref	-
Infected	0.63 (0.46 – 0.87)	0.005	1.02 (0.61 – 1.68)	0.95
**Perceived risk of hospitalisation due to COVID-19** (scale 0 – 100), per 10 point increase	1.06 (1.02 – 1.10)	0.003	1.16 (1.08 – 1.24)	<0.001
**Perceived risk of spreading COVID-19**				
Not at all likely	Ref	-	Ref	-
A little likely	0.96 (0.70 – 1.33)	0.82	1.15 (0.65 – 2.03)	0.64
Quite likely	0.89 (0.63 – 1.25)	0.50	1.33 (0.73 – 2.41)	0.35
Very likely	0.78 (0.56 – 1.09)	0.15	1.58 (0.89 – 2.80)	0.12
**Cohabitation**				
Does not live with someone over 65 years old	Ref	-	Ref	-
Lives with someone over 65 years old	0.90 (0.64 – 1.28)	0.57	1.15 (0.64 – 2.08)	0.64

Supplementary Table 7 shows the results of multivariable logistic regression as adjusted odds ratios, 95% confidence intervals and p values for the association of sociodemographic, occupational and perceived risk variables with outcome 3 (work changed as a result of risk assessment) and outcome 4 (work did not change, but changes were wanted). Odds ratios are adjusted for age, sex, ethnicity, occupation and migration status. We did not include the 'perceived risk' variables in the analysis of outcome 1 as we felt these would not have influenced whether a HCW was offered a risk assessment.

*In the occupation variable, nursing includes midwives, nursing associates and healthcare assistants; allied health professionals includes pharmacists, healthcare scientists, ambulance workers and those in optical roles.

BMI - Body mass index; COVID-19 - coronavirus disease 2019; SARS-CoV-2 - Severe acute respiratory syndrome coronavirus-2

Supplementary information

Supplementary Table 6. Adjusted analysis of outcomes 1 and 2 in complete cases

	OUTCOME 1 – OFFERED A RISK ASSESSMENT (n=6,767)		OUTCOME 2 – COMPLETED A RISK ASSESSMENT (n=5,503)	
	aOR (95%CI)	P value	aOR (95%CI)	P value
**Ethnicity**				
White	Ref	-	Ref	-
Asian	2.35 (1.89 – 2.91)	<0.001	2.79 (1.95 – 3.99)	<0.001
Black	2.37 (1.54 – 3.66)	<0.001	3.00 (1.43 – 6.30)	0.004
Mixed	2.31 (1.56 – 3.41)	<0.001	1.40 (0.85 – 2.31)	0.19
Other	1.47 (0.90 – 2.40)	0.13	1.42 (0.68 – 2.95)	0.35
**Age,** per decade increase	1.03 (0.97 – 1.08)	0.39	1.17 (1.06 – 1.28)	0.001
**Sex**				
Male	Ref	-	Ref	-
Female	1.16 (1.00 – 1.35)	0.06	1.47 (1.15 – 1.87)	0.002
**Occupation***				
Medical	Ref	-	Ref	-
Nursing	1.13 (0.92 – 1.37)	0.24	1.50 (1.08 – 2.07)	0.02
Allied Health Professional	1.12 (0.94 – 1.33)	0.19	1.40 (1.07 – 1.83)	0.02
Dental	0.60 (0.43 – 0.82)	0.002	3.23 (1.29 – 8.07)	0.01
Admin/estates/other	1.53 (1.08 – 2.16)	0.02	1.42 (0.85 – 2.37)	0.18
**Migration status**				
Born in the UK	Ref	-	Ref	-
Born overseas	1.24 (1.03 – 1.48)	0.02	1.08 (0.81 – 1.45)	0.58
**Exposure to COVID-19 patients**				
None (or remote only)	Ref	-	Ref	-
Face-to-face with social distancing	1.21 (0.89 – 1.65)	0.23	0.55 (0.37 – 0.82)	0.004
Physical contact	0.74 (0.64 – 0.85)	<0.001	0.73 (0.57 – 0.93)	0.009
**Number of long-term physical health conditions**				
0	Ref	-	Ref	-
1	1.44 (1.22 – 1.71)	<0.001	1.71 (1.27 – 2.30)	<0.001
≥2	2.06 (1.43 – 2.97)	<0.001	1.92 (1.05 – 3.49)	0.03
**BMI (kg/m^2)**				
< 25	Ref	-	Ref	-
≥ 25 to < 30	0.93 (0.81 – 1.08)	0.37	0.91 (0.71 – 1.16)	0.45
≥ 30	1.03 (0.87 – 1.23)	0.70	1.02 (0.76 – 1.35)	0.91
**SARS-CoV-2 infection status**				
Uninfected	Ref	-	Ref	-
Infected	0.80 (0.67 – 0.96)	0.01	0.83 (0.62 – 1.11)	0.20
**Perceived risk of hospitalisation due to COVID-19** (scale 0 – 100), per 10 point increase	-	-	1.08 (1.03 – 1.14)	0.002
**Perceived risk of spreading COVID-19**				
Not at all likely	-	-	Ref	-
A little likely			1.18 (0.85 – 1.63)	0.32
Quite likely			1.31 (0.93 – 1.85)	0.13
Very likely			1.45 (1.00 – 2.10)	0.049
**Cohabitation**				
Does not live with someone over 65 years old	-	-	Ref	-
Lives with someone over 65 years old			0.77 (0.50 – 1.16)	0.21

Supplementary Table 8 shows the results of multivariable logistic regression as adjusted odds ratios, 95% confidence intervals and p values for the association of sociodemographic, occupational and perceived risk variables with outcome 1 (offered a risk assessment) and outcome 2 (completed a risk assessment once offered). Odds ratios are adjusted for age, sex, ethnicity, occupation and migration status. Exclusions for each outcome are the same as the main analysis with the addition of exclusion of those with missing data in any of the variables of interest.

*In the occupation variable, nursing includes midwives, nursing associates and healthcare assistants; allied health professionals includes pharmacists, healthcare scientists, ambulance workers and those in optical roles.

BMI - Body mass index; COVID-19 - coronavirus disease 2019; SARS-CoV-2 - Severe acute respiratory syndrome coronavirus-2

Supplementary information

Supplementary Table 7. Adjusted analysis of outcomes 3 and 4 in complete cases

	OUTCOME 3 – WORKING PRACTICES CHANGED (n=1,492)		OUTCOME 4 – WANTED CHANGES BUT WORK DIDN'T CHANGE (n=442)	
	**aOR (95%CI)**	**P value**	**aOR (95%CI)**	**P value**
**Ethnicity**				
White	Ref	-	Ref	-
Asian	0.49 (0.35 – 0.68)	<0.001	2.02 (1.17 – 3.49)	0.01
Black	0.60 (0.36 – 1.01)	0.05	2.83 (1.19 – 6.74)	0.02
Mixed	0.47 (0.30 – 0.74)	0.001	1.36 (0.66 – 2.81)	0.41
Other	0.34 (0.16 – 0.71)	0.004	1.25 (0.40 – 3.95)	0.70
**Age,** per decade increase	1.25 (1.13 – 1.39)	<0.001	0.88 (0.74 – 1.06)	0.17
**Sex**				
Male	Ref	-	Ref	-
Female	1.27 (0.96 – 1.67)	0.09	1.74 (1.11 – 2.70)	0.02
**Occupation**				
Medical	Ref	-	Ref	-
Nursing	1.24 (0.87 – 1.77)	0.23	1.89 (1.06 – 3.38)	0.03
Allied Health Professional*	1.47 (1.09 – 2.00)	0.01	1.52 (0.92 – 2.49)	0.10
Dental	1.36 (0.72 – 2.57)	0.34	3.63 (1.16 – 11.35)	0.03
Admin/estates/other	3.71 (1.69 – 8.15)	0.001	4.70 (0.87 – 25.3)	0.07
**Migration status**				
Born in the UK	Ref	-	Ref	-
Born overseas	1.14 (0.85 – 1.53)	0.39	0.93 (0.58 – 1.51)	0.78
**Exposure to COVID-19 patients**				
None (or remote only)	Ref	-	Ref	-
Face-to-face with social distancing	0.44 (0.27 – 0.71)	0.001	0.47 (0.21 – 1.09)	0.08
Physical contact	0.35 (0.27 – 0.46)	<0.001	0.53 (0.34 – 0.82)	0.005
**Number of long-term physical health conditions**				
0	Ref	-	Ref	-
1	1.05 (0.82 – 1.35)	0.68	0.51 (0.33 – 0.79)	0.002
≥2	1.69 (1.15 – 2.48)	0.008	0.79 (0.39 – 1.63)	0.53
**BMI (kg/m^2)**				
< 25	Ref	-	Ref	-
≥ 25 to < 30	1.03 (0.78 – 1.35)	0.84	1.30 (0.82 – 2.08)	0.27
≥ 30	1.02 (0.76 – 1.36)	0.91	1.16 (0.69 – 1.93)	0.58
**SARS-CoV-2 infection status**				
Uninfected	Ref	-	Ref	-
Infected	0.58 (0.41 – 0.82)	0.002	0.73 (0.41 – 1.29)	0.28
**Perceived risk of hospitalisation due to COVID-19** (scale 0 – 100), per 10 point increase	1.06 (1.02 – 1.11)	0.005	1.15 (1.06 – 1.24)	<0.001
**Perceived risk of spreading COVID-19**				
Not at all likely	Ref	-	Ref	-
A little likely	0.96 (0.67 – 1.38)	0.84	1.23 (0.65 – 2.32)	0.52
Quite likely	0.85 (0.58 – 1.25)	0.41	1.36 (0.70 – 2.62)	0.36
Very likely	0.85 (0.59 – 1.25)	0.42	1.43 (0.74 – 2.77)	0.29
**Cohabitation**				
Does not live with someone over 65 years old	Ref	-	Ref	-
Lives with someone over 65 years old	0.96 (0.63 – 1.47)	0.86	1.03 (0.49 – 2.15)	0.94

Supplementary Table 9 shows the results of multivariable logistic regression as adjusted odds ratios, 95% confidence intervals and p values for the association of sociodemographic, occupational and perceived risk variables with outcome 3 (work changed as a result of risk assessment) and outcome 4 (work did not change, but changes were wanted). Odds ratios are adjusted for age, sex, ethnicity, occupation and migration status. Exclusions for each outcome are the same as the main analysis with the addition of exclusion of those with missing data in any of the variables of interest.

*In the occupation variable, nursing includes midwives, nursing associates and healthcare assistants; allied health professionals includes pharmacists, healthcare scientists, ambulance workers and those in optical roles.

BMI - Body mass index; COVID-19 - coronavirus disease 2019; SARS-CoV-2 - Severe acute respiratory syndrome coronavirus-2

Supplementary information

Supplementary Table 8. Univariable logistic regression to show the association of ethnic group with risk assessment coverage and outcomes

	**OUTCOME 1 – OFFERED A RISK ASSESSMENT N=8,649**		**OUTCOME 2 – COMPLETED A RISK ASSESSMENT N=7,083**		**OUTCOME 3 – WORKING PRACTICES CHANGED N=1,964**		**OUTCOME 4 – WANTED CHANGES BUT WORK DIDN'T CHANGE N=579**	
	**Odds ratio (95% CI)**	**P value**	**Odds ratio (95% CI)**	**P value**	**Odds ratio (95% CI)**	**P value**	**Odds ratio (95% CI)**	**P value**
**Ethnicity**								
White	Ref	-	Ref	-	Ref	-	Ref	-
Asian	2.33 (1.98 – 2.73)	<0.001	1.90 (1.48 – 2.46)	<0.001	0.44 (0.36 – 0.55)	<0.001	1.31 (0.91 – 1.89)	0.14
Black	3.23 (2.20 – 4.75)	<0.001	2.12 (1.23 – 3.66)	0.007	0.53 (0.36 – 0.79)	0.002	1.70 (0.89 – 3.25)	0.11
Mixed	1.97 (1.43 – 2.72)	<0.001	1.21 (0.78 – 1.89)	0.40	0.45 (0.30 – 0.66)	<0.001	0.91 (0.49 – 1.70)	0.78
Other	1.48 (1.00 – 2.18)	0.049	1.07 (0.60 – 1.91)	0.81	0.37 (0.21 – 0.66)	0.001	0.88 (0.36 – 2.17)	0.78

Supplementary information

Supplementary Table 9. Analysis of the relationship between ethnicity and risk assessment coverage and outcomes in staff who reported contact with COVID patients during lockdown

	OUTCOME 1 – OFFERED A RISK ASSESSMENT N=4,458		OUTCOME 2 – COMPLETED A RISK ASSESSMENT N=3,591		OUTCOME 3 – WORKING PRACTICES CHANGED N=851		OUTCOME 4 – WANTED CHANGES BUT WORK DIDN'T CHANGE N=361	
	Adjusted odds ratio (95% CI)	P value	Adjusted odds ratio (95% CI)	P value	Adjusted odds ratio (95% CI)	P value	Adjusted odds ratio (95% CI)	P value
**Ethnicity**								
White	Ref	-	Ref	-	Ref	-	Ref	-
Asian	2.52 (1.95 – 3.27)	<0.001	2.04 (1.40 – 2.98)	<0.001	0.63 (0.42 – 0.95)	0.03	2.53 (1.28 – 5.01)	0.008
Black	3.80 (2.23 – 6.47)	<0.001	1.92 (1.02 – 3.62)	0.04	0.76 (0.44 – 1.33)	0.35	3.32 (1.34 – 8.21)	0.009
Mixed	2.28 (1.47 – 3.55)	<0.001	1.36 (0.76 – 2.41)	0.30	0.45 (0.25 – 0.82)	0.009	1.05 (0.45 – 2.45)	0.92
Other	1.43 (0.86 – 2.37)	0.17	0.90 (0.47 – 1.72)	0.75	0.56 (0.24 – 1.31)	0.18	1.76 (0.50 – 6.14)	0.38

Supplementary Table 11 shows the relationship between ethnicity and our outcome measures after exclusion of those who reported no direct contact with COVID-19 patients and after adjustment for age, sex, occupation and migration status. It should be noted that due to lower numbers of participants included in outcomes 3 and 4 and the overlap between direct patient contact and particular healthcare roles the occupation variable was collapsed into fewer categories for these analyses.

Supplementary information

Supplementary Figure 1. Recruitment

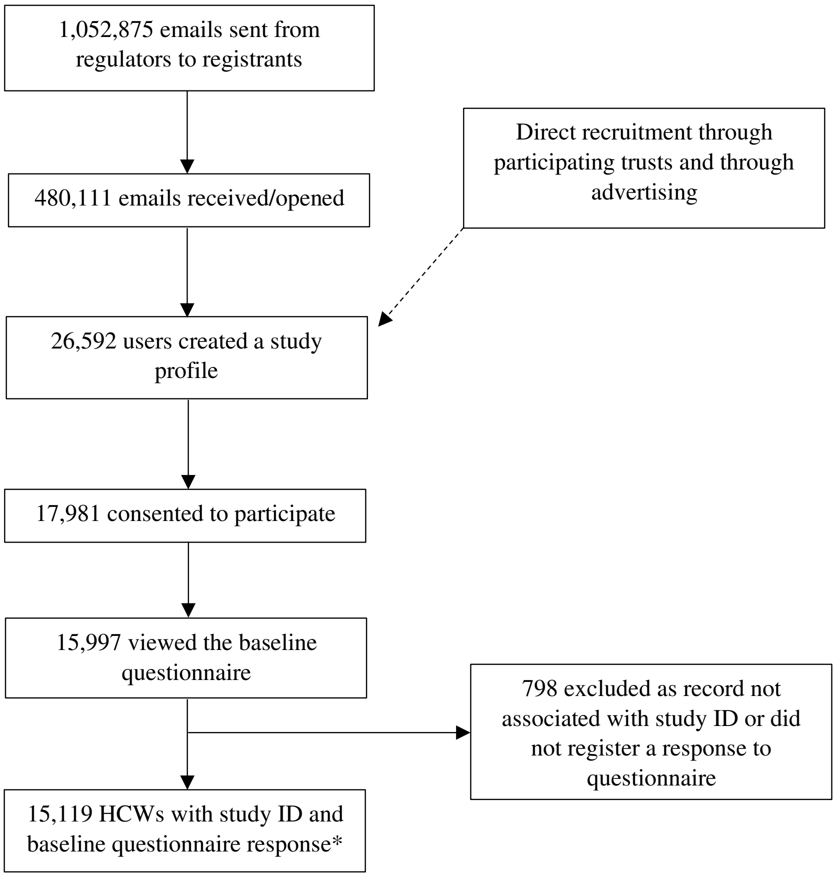

*corresponds to a response rate of 57.1% of those who registered/created a profile on the study website (and 84.5% of those who consented, 1.4% of those who were sent an email and 3.2% of those who opened the email).

For further details on the formation of the analysed sample, see Figure 1. HCW – healthcare worker.
